# Tissue Harvesting Site Effect on the Canine Adipose Stromal Vascular Fraction Quantity and Quality

**DOI:** 10.3390/ani11020460

**Published:** 2021-02-09

**Authors:** Hanan Hendawy, Akiko Uemura, Danfu Ma, Ryosuke Namiki, Haney Samir, Mahmoud F. Ahmed, Ahmed Elfadadny, Hussein M. El-Husseiny, Cheng Chieh-Jen, Ryou Tanaka

**Affiliations:** 1 Laboratory of Veterinary Surgery, Tokyo University of Agriculture and Technology, Tokyo 183-8509, Japan; hanan_attia@vet.suez.edu.eg (H.H.); anco@vet.ne.jp (A.U.); dandanma1000@gmail.com (D.M.); nxwcm554@ybb.ne.jp (R.N.); s195162s@st.go.tuat.ac.jp (H.M.E.-H.); john_199328@yahoo.com.tw (C.C.-J.); 2 Department of Veterinary Surgery, Faculty of Veterinary Medicine, Suez Canal University, Ismailia 41522, Egypt; mahmoud_ali@vet.suez.edu.eg; 3 Department of Theriogenology, Faculty of Veterinary Medicine, Cairo University, Giza 12211, Egypt; haneyvet360@yahoo.com; 4 Laboratory of Veterinary Physiology, Department of Veterinary Medicine, Tokyo University of Agriculture and Technology, Tokyo 183-8509, Japan; 5 Department of Animal Medicine, Faculty of Veterinary Medicine, Damanhur University, Damanhur, El-Beheira 22511, Egypt; ahmed.elfadadny@vetmed.dmu.edu.eg; 6 Department of Surgery, Anesthesiology, and Radiology, Faculty of Veterinary Medicine, Benha University, Elqaliobiya 13736, Egypt

**Keywords:** canine, stromal vascular fraction, harvest site, adipose-derived MSCs, flow cytometry

## Abstract

**Simple Summary:**

Adipose stromal vascular fraction (SVF) cells are freshly isolated non-cultured mesenchymal stem cells, which have been recently applied in the treatment of several musculoskeletal inflammatory conditions in dogs. However, the best adipose tissue (AT) sampling site is still challenging. This study first addressed the ideal AT harvesting site in canines ranging between middle and old age, the most susceptible age to chronic musculoskeletal problems. Our results showed that the peri-ovarian region is the best AT harvesting site, which yields high amounts of SVF cells with enough adipose-derived stem cells. These data may help the further set-up of cell-based regenerative therapies at the preclinical and experimental level in canines.

**Abstract:**

Mesenchymal stem cells (MSCs) constitute a great promise for regenerative therapy, but these cells are difficultly recovered in large amounts. A potent alternative is the stromal vascular fraction (SVF), non-cultured MSCs, separated from adipose tissue (AT). We aim to evaluate AT harvesting site effect on the SVF cells’ quantity and quality in dogs. Subcutaneous abdominal fat, falciform ligament and peri-ovarian fat were sampled. After SVF isolation, the trypan blue exclusion test and a hemocytometer were used to assess the cell viability and cellular yield. SVF cells were labeled for four surface antigenic markers, clusters of differentiation CD90, CD44, CD29, and CD45, and then examined by flow cytometry. Semi-quantitative RT-PCR was used to evaluate the gene expression of the former markers in addition to OCT-4 and CD34. SVF cells in the peri-ovarian AT recorded the highest viability% (99.63 ± 0.2%), as well as a significantly higher cellular yield (36.87 ± 19.6 × 10^6^ viable cells/gm fat, *p* < 0.001) and a higher expression of adipose-derived mesenchymal stem cells AD-MSCs surface markers than that of other sites. SVF cells from the peri-ovarian site revealed a higher expression of MSC markers (CD90, CD44, and CD29) and OCT-4 compared to the other sites, with weak CD45 and CD34 expressions. The positive OCT-4 expression demonstrated the pluripotency of SVF cells isolated from different sites. To conclude, the harvesting site is a strong determinant of SVF cells’ quantity and quality, and the peri-ovarian site could be the best AT sampling site in dogs.

## 1. Introduction

The use of mesenchymal stem cells (MSCs) in regenerative medicine holds a great promise for repairing damaged tissues in both acute conditions, such as injuries of ligaments, tendons, cartilage or bone, and chronic conditions, such as osteoarthritis [1]. These cells have been separated from many tissues, including bone marrow, adipose tissue, blood, cartilage, and muscle [2]. Although studies initially focused on bone marrow-derived MSCs, the high concentration of MSCs in adipose tissues (100–1000 times that in bone marrow) triggered adipose-derived MSCs’ use in regenerative stem cell therapy [3,4,5]. Adipose tissue (AT) is a practical and reasonable source for both freshly isolated stromal vascular fraction and cultured adipose-derived stem cells (AD-MSCs) [6]. Stromal vascular fraction (SVF) cells can be directly separated from minced AT tissue by incubation with collagenase enzyme followed by centrifugation [7,8]. SVF cells are a heterogeneous cell population from adipose stromal cells, progenitor cells, hematopoietic stem cells, and endothelial cells. This cell population produces a homogeneous cell population of plastic adherent AD-MSCs when expanded on culture [9]. Both cell types, primary SVF and cultured AD-MSCs, represent an important therapeutic target [10].

Recent clinical trials have proposed using freshly isolated primary SVF cells instead of cultured AD-MSCs [11,12,13] for many reasons. Cultivation of the SVF cells leads to changes in the AD-MSCs’ phenotype and reduces the differentiation potential of these cells. Thus, the primary SVF cells have a larger regenerative capacity than cultured AD-MSCs [14,15]. Additionally, the popularity of freshly isolated SVF cells over cultured AD-MSCs in the veterinary practice may be due to the high-expenses, time-consuming isolation and the in vitro serial expansion of AD-MSCs, which may lead to contamination, loss of differentiation ability, and the neoplastic transformation of cells [6]. It is beneficial to isolate autologous SVF cells and re-use them as a therapy in a single surgical procedure. Therefore, SVF cells may have the chief prospective for future stem cell therapy [12].

Dogs are a good preclinical animal model to study several degenerative and traumatic diseases in humans [16,17]. Recently, adipose SVF has been used for the treatment of several inflammatory and immune-mediated conditions in canines, such as osteoarthritis, tendinopathy, hip dysplasia and multiple sclerosis [10,11,18,19]. Despite the wide use of canine SVF cells in veterinary clinics, only two studies investigated the cellular component of freshly isolated canine SVF cells. Astor et al. [20] discussed AT collection from three different sites (subcutaneous caudal to the scapula, falciform, and inguinal region). However, Astor et al. [20] did not assess the immune phenotyping of the freshly isolated SVF cells. Sullivan et al. [21] conducted another study, in which dogs were less than 2 years of age, and AT samples were collected from two sites only (subcutaneous AT caudal to the scapula and falciform ligament). However, Astor et al. [20] and Sullivan et al. [21] did not investigate the peri-ovarian site as a promising source for AT harvest. Despite this expansion, SVF cells have not been characterized in dogs compared to those reports in humans or bone marrow cells [21]. Our study aims to investigate the best AT harvesting site in middle-aged to older dogs regarding the quantity and quality of the SVF cells. Thus, we isolated AT from abdominal subcutaneous fat, falciform ligament, and peri-ovarian sites. Then, we compared the isolated SVF cells in cell viability, cellular yield, and AD-MSC surface markers’ expression using the trypan blue exclusion test, a hemocytometer, and flow cytometry. Flow cytometry results were validated by semi-quantitative RT-PCR.

## 2. Materials and Methods

### 2.1. Ethical Statement

All experiments were compliant with ethical standards and safety guidelines of regenerative medicine and cell therapy in dogs and cats designed by the Japanese Society for Veterinary Regenerative Medicine. We had extracted adipose tissue samples from dogs only after their owners provided written informed consent.

### 2.2. Animals

This study involved ten healthy female dogs that were admitted to the Animal Medical Center at Tokyo University of Agriculture and Technology, Japan for spaying. As the most susceptible age to chronic musculoskeletal problems, middle to old age dogs (mean 8.9 ± 1.1 years) were used for AT extraction, with the mean body weight of 9.55 ± 4.86 kg. These chosen dogs belonged to different breeds: Beagle (*n* = 2), Chihuahua (*n* = 2), Miniature Dachshund (*n* = 1), Pug (*n* = 1), Golden Retriever (*n* = 1), Jack Russell Terrier (*n* = 1), Pomeranian (*n* = 1), and Mix (*n* = 1). All dogs were examined for complete blood count, urine analysis, and serum biochemistry. Before extracting AT samples, dog anesthesia was initiated by I/V injection of Propofol (6 mg/kg, Propofol 1%, Nichi-Iko, Toyama, Japan) and maintained by 2% isoflurane (Isoflu; Dainippon Sumitomo Pharma, Chuo-ku, Osaka, Japan) intubation [22,23]. Then, the surgical field was aseptically prepared and draped.

### 2.3. AT Harvesting

AT samples were collected from three different sites: abdominal subcutaneous, falciform ligament, and the peri-ovarian region. Subcutaneous AT was extracted from surgical wound edges, while the falciform ligament fats were immediately harvested after a midline celiotomy incision. The peri-ovarian region AT was collected from the uterine broad ligament-enclosing fats by monopolar electrocautery according to the standard surgical technique. All dogs were further monitored for any surgical complications.

### 2.4. Isolation of SVF Cells

We isolated SVF cells from AT samples under complete aseptic conditions using procedures described by Zuk et al. [5], with little modifications. Briefly, AT samples were collected in 50 mL conical tubes (Falcon^®^, Corning Inc., Tewksbury, MA, USA) and weighed. Extensive washing was performed with phosphate-buffered saline (PBS). Then, tissues were placed in sterile dishes and minced into small pieces (1–3 mm) with a sterile scalpel. A 0.2% collagenase (Gibco; Thermo Fisher Scientific, Inc., Waltham, MA, USA)/Hanks’ Balanced Salt Solution (HBSS; Wako Pure Chemical Industries, Ltd., Chuo-ku, Osaka, Japan) mixture (mL) was added to the minced AT (cm^3^) at the ratio of 1:1 and incubated at 37 °C with shaking (120 rpm, 30 min). The enzymatic activity was neutralized by cold HBSS. Following centrifugation (800× *g*, 10 min), the cell pellet containing SVF cells was collected, rewashed with HBSS, and successively filtered using 100 μm and 40 μm nylon meshes to remove any cellular debris. Freshly isolated SVF cells were used in assessing the cell viability and quantity and in flow cytometry. However, the remaining cells were suspended in a freezing medium, containing 10% Dulbecco’s Modified Eagle’s medium (DMEM/F-12, Gibco; Thermo Fisher Scientific, Inc.), 10% dimethyl sulfoxide (DMSO, Wako Pure Chemical Industries, Ltd.), and 80% fetal bovine serum (FBS, Gibco; Thermo Fisher Scientific, Inc.) and cryopreserved at −80 °C using a Bicell cryopreservation device (Nihon-freezer, Tokyo, Japan) until use for RT-PCR.

### 2.5. Assessment of SVF Cells Viability and Quantity

Cell viability and quantity were determined using a hemocytometer combined with the routine trypan blue exclusion test. SVF cell suspension (10 μL) was diluted 1:1 with 0.4% trypan blue solution (Gibco; Thermo Fisher Scientific, Inc., Waltham, MA, USA) and loaded into the hemocytometer chamber. We used the average of two full squares to calculate the percentage of viable cells. SVF cellular yield or concentration was calculated by dividing the total number of viable cells per gm fat (dry fat digested by collagenase). Data were represented as the number of viable cells × 10^6^/gm of dry fat ± standard deviation [1].

### 2.6. Immunophenotyping of the Potential AD-MSCs in SVF Samples

We used flow cytometry to assess the potential of AD-MSC subpopulations from the freshly isolated SVF samples from different AT extraction sites. SVF cells were labeled with a panel of monoclonal antibodies against mesenchymal (CD90, CD44, and CD29) and hematopoietic (CD45) stem cell markers according to Krešić et al. [24] and Yaneselli et al. [25]. Briefly, SVF cells were suspended in PBS and incubated at 4 °C for 30 min with Phycoerythrin (PE)-conjugated antibodies against CD90, CD44, and CD29, and fluorescein isothiocyanate (FITC)-conjugated antibody against CD45, listed in Table 1. Flow cytometry analyses were performed using the CytoFLEX Flow Cytometer (Beckman Coulter, Brea, CA, USA) equipped with a blue laser (488 nm). The percentage of each marker was separately detected in SVF samples. The resulting data were further analyzed using CytExpert Software v1.2.

### 2.7. Semi-Quantitative RT-PCR

The phenotypic expression of different MSC markers was assessed by performing Reverse-Transcriptase-PCR (RT-PCR) as previously described [26]. Table 2 shows the details of analyzed genes and the sequence of specific primers. All primers were manufactured by the FASMAC Company, Midorigaoka, Kanagawa, Japan. Total RNA was extracted from SVF cells (1.5 × 10^6^) isolated from different harvesting sites using RNeasy Isolation Kit (Qiagen AG, Garstligweg, Hombrechtikon, Switzerland) with DNase I treatment following the manufacturer’s protocol. The RNA quantity was measured by a NanoDrop ND-1000 spectrophotometer (NanoDropTechnologies, Wilmington, NC, USA). Agarose gel electrophoresis was used to assess the RNA integrity. First-strand complementary DNA cDNAwas synthesized from 1 μg of total RNA with a PrimeScript RT Master Mix (Takara, Kusatsu, Shiga, Japan) according to the manufacturer’s protocol. EmeraldAmp MAX PCR Master Mix (Takara, Kusatsu, Shiga, Japan) was used for RT-PCR. The final RT-PCR mixtures contained 25 µL EmeraldAmp MAX PCR Master Mix, 2 µL template cDNA, 0.2 µM of each specific forward and reverse primer, and ddH_2_O up to 50 µL. Cycling protocols were as follows: 35 cycles of denaturation at 94 °C for 30 s, annealing at 55 °C for 30 s, and extension at 72 °C for 1 min. RT-PCR products were examined on ethidium bromide-stained agarose gel (1.5%) in TAE (Tris Acetate-EDTA) buffer. Amplicons were visualized using a UV Transilluminator, and images were captured by a Canon digital camera. All analyses were repeated with two replicates for each AD harvesting site sample. For semi-quantitative analysis of MSC markers expression, we used the ImageJ image processing software to evaluate the optical density of each positive band normalized to that of the endogenous housekeeping gene (*β*-actin).

### 2.8. Statistical Analysis

All data were expressed as mean ± standard deviation values (SD). Statistical analysis was carried out using GraphPad Prism software version 6 (GraphPad Software, Inc., La Jolla, CA, USA). Data were analyzed by one-way analysis of variance (ANOVA) followed by Tukey’s post hoc test to evaluate differences between groups. Statistically significant differences were considered at a *p* value less than 0.05.

## 3. Results

### 3.1. Effect of Harvesting Site on AT yield

Table 3 shows samples’ weights for each adipose tissue submission. The mean weight of collected AT samples was 2.7 ± 1.6, 9.47 ± 2.9, and 8.13 ± 4.3 gm for the subcutaneous abdominal, falciform ligament, and peri-ovarian AT, respectively. Compared to the subcutaneous abdominal and falciform ligament sites, the recovered AT weight was highly variable at the peri-ovarian site. Moreover, AT harvesting from both falciform ligament and peri-ovarian sites was much easier than that of subcutaneous abdominal AT harvesting, which required an extensive dissection. No clinical complications were recorded in dogs either during or after AT harvesting from all sites.

### 3.2. Effect of AT Harvesting Site on SVF Cell Viability and Quantity

**Figure 1:**
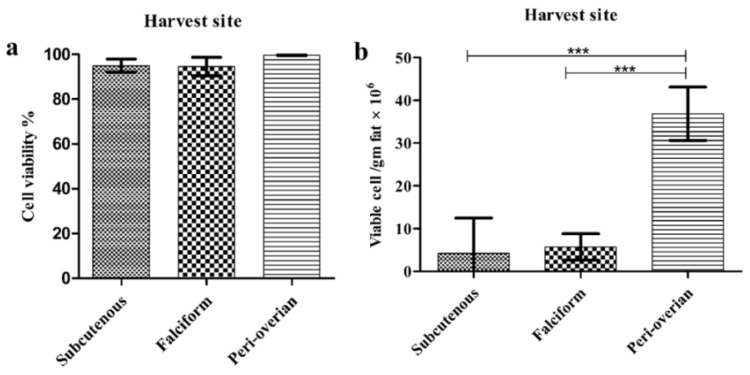
The effect of harvesting site on stromal vascular fraction (SVF) cell viability and quantity. (**a**) Viability percentage of the SVF cells from different harvesting sites. SVF cells isolated from per-ovarian adipose tissue (AT) showed the highest viability percentage; (**b**) Number of viable cells per gram fat from different harvesting sites. SVF cells isolated from the per-ovarian AT showed the highest number of viable cells per gram fat at *** *p*< 0.001. Data are expressed as the mean ± standard deviation (*n* = 10). Statistical significance was tested using one-way ANOVA followed by Tukey’s post hoc test for multi-group comparisons. *** *p* < 0.001.

Cell viability was examined after the extraction of SVF cells from different sites. Among all, SVF cells isolated from peri-ovarian AT showed the highest viability percentage, but without significant difference. The mean SVF cell viability% was 94.94 ± 2.9, 94.58 ± 4.1, and 99.63 ± 0.2% for the subcutaneous abdominal, falciform ligament, and peri-ovarian AT, respectively (Figure 1a). We did not find any significant difference in the number of viable cells/gm fat between the falciform ligament and subcutaneous abdominal sites. However, peri-ovarian AT showed the highest number of viable cells per gram fat (36.87 ± 19.6 × 10^6^) at a significant level (*p* < 0.001) compared to the subcutaneous abdominal site (4.18 ± 8.25 × 10^6^) and the falciform ligament site (5.71 ± 3.09 × 10^6^), as shown in Figure 1b.

### 3.3. The Potential AD-MSCs in the Freshly Isolated SVF Cells from Different Harvesting Sites

**Figure 2:**
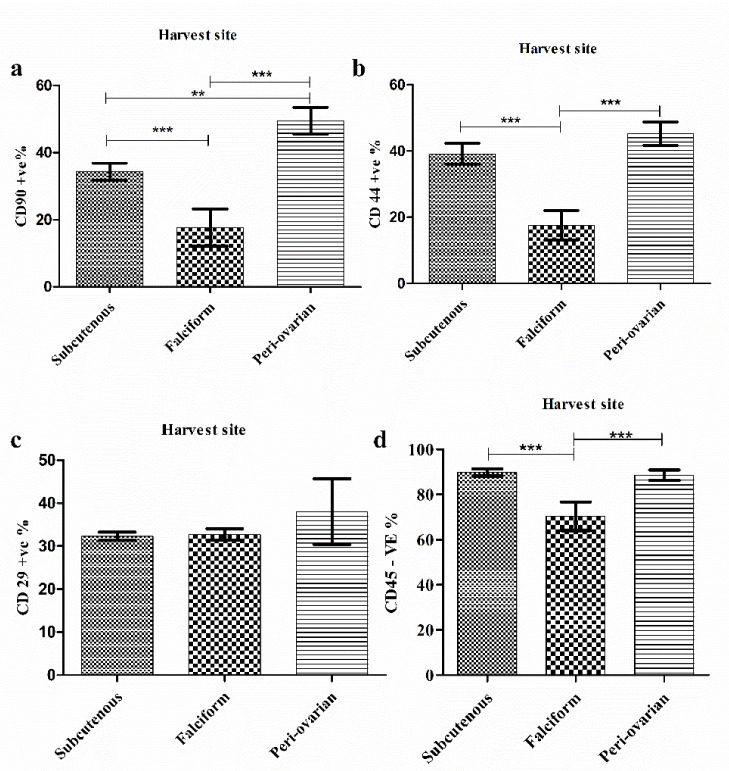
The effect of harvesting site on the mean percentage of adipose-derived mesenchymal stem cells surface marker expression in SVF cells. (**a**) The mean percentage of CD90 positive cells from different harvesting sites; (**b**) The mean percentage of CD44 positive cells from different harvesting sites; (**c**) The mean percentage of CD29 positive cells from different harvesting sites; (**d**) The mean percentage of CD45 negative cells from different harvesting sites. Data are represented as the mean percentage ± standard deviation (*n* = 10). ** *p*< 0.01: a significant difference; *** *p*< 0.001: a highly significant difference.

We assessed the harvesting site effect on the phenotype of the potential AD-MSCs within the SVF cells by calculating the mean percentage of cells positive to MSCs markers and negative to the hematopoietic marker. The percentage of SVF cells with CD90^+^ve expression for the peri-ovarian site (49.56 ± 4.9%) was significantly higher *(p* < 0.01) than that of the abdominal and falciform ligament (34.32 ± 2.55%, 17.65 ± 5.52%, respectively) (Figure 2a). Similarly, the highest percentage of CD44+ve cells was recorded in the peri-ovarian SVF cells (45.25 ± 3.55%) (Figure 2b). CD29+ve cells showed no significant differences among the three sites and were 32.34 ± 0.94% for the subcutaneous abdominal site, 32.68 ± 0.8% for the falciform ligament, and 38.00 ± 62.7% for the peri-ovarian site (Figure 2c). To confirm cell identity, the CD45 surface marker was used to identify hematopoietic cell contamination (Figure 2d). The percentages of CD45-ve SVF cells were significantly higher (*p* < 0.001) in the cells obtained from the subcutaneous abdominal site (89.77 ± 1.62%) and the peri-ovarian site (88.58 ± 2.25%) than those recovered from the falciform ligament site (70.35 ± 6.33%).

**Figure 3:**
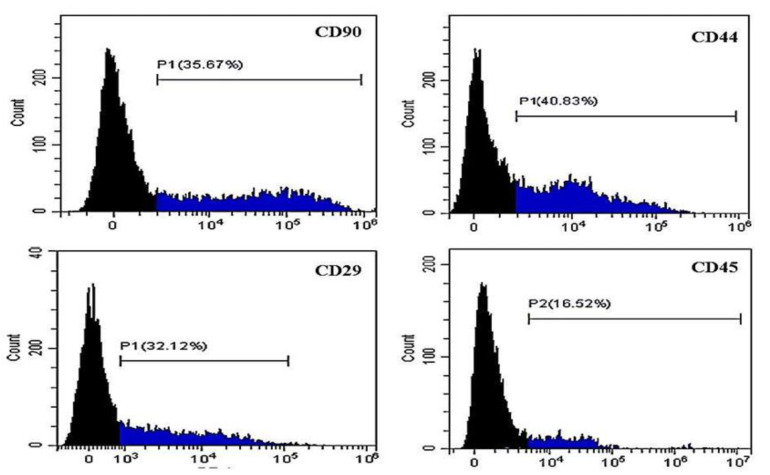
Results of flow cytometry assay of canine SVF cells isolated from the subcutaneous abdominal AT stained with PE-conjugated anti-CD90, anti-CD44, anti-CD29, and FITC-conjugated anti-CD45. Black areas indicate unstained cells. The percentage represents gated positive areas.

**Figure 4:**
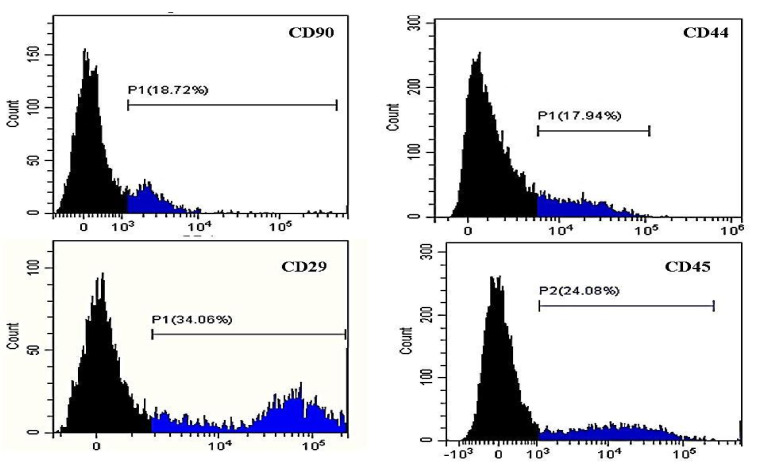
Results of flow cytometry assay of canine SVF cells isolated from the falciform ligament AT stained with PE-conjugated anti-CD90, anti-CD44, anti-CD29, and FITC-conjugated anti-CD45. Black areas indicate negative areas. The percentage represents gated positive areas.

**Figure 5:**
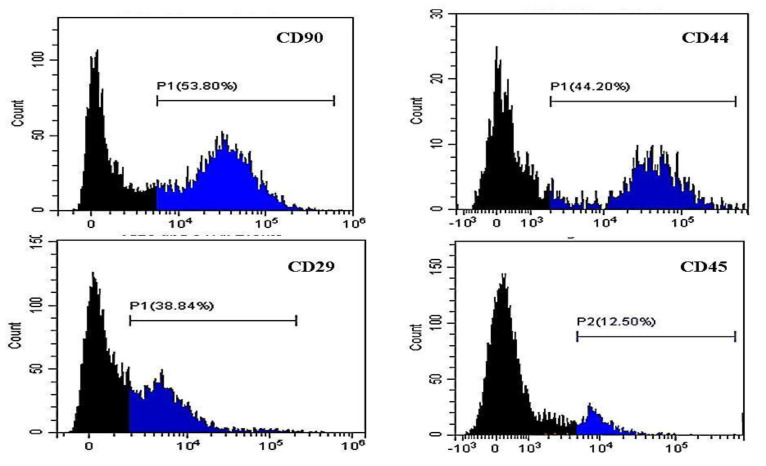
Results of flow cytometry assay of canine SVF cells isolated from the peri-ovarian AT stained with PE-conjugated anti-CD90, anti-CD44, anti-CD29, and FITC-conjugated anti-CD45. Black areas indicate unstained cells. The percentage represents gated positive areas.

Figure 3, Figure 4 and Figure 5 show representative histograms for using the flow cytometer to detect the potential AD-MSCs within the SVF cells isolated from the subcutaneous abdominal, falciform ligament, and peri-ovarian AT, respectively.

### 3.4. Semi-Quantitative RT-PCR Results

**Figure 6:**
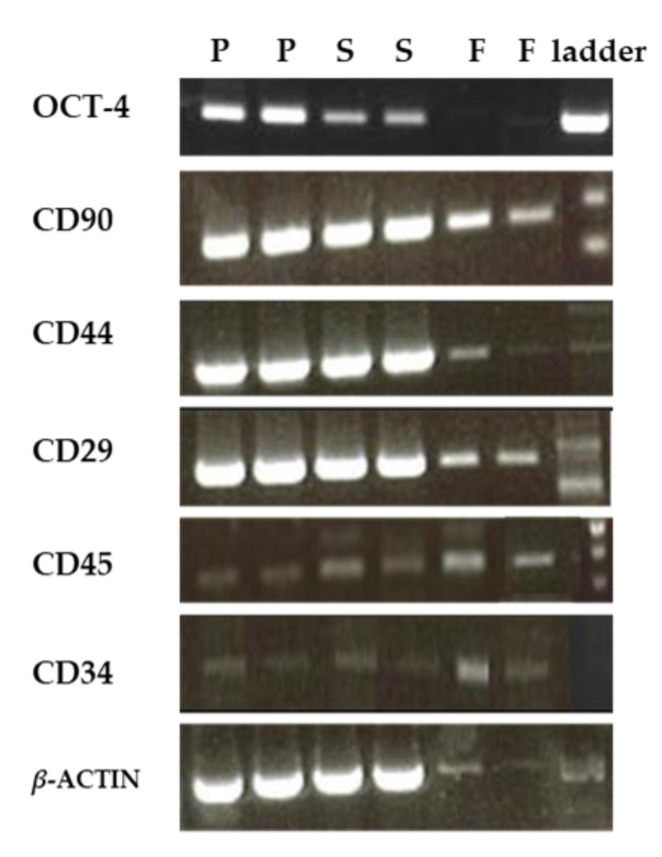
RT-PCR analysis of OCT-4, CD90, CD44, CD29, CD45, CD34, and *β*-ACTIN genes in SVF cells isolated from the peri-ovarian (P), subcutaneous abdominal (S), and falciform ligament (F) fats.

**Figure 7:**
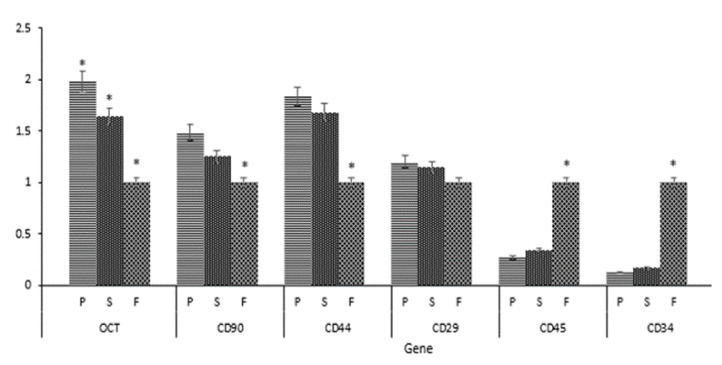
Semi-quantitative data from the gels using ImageJ software. P: peri-ovarian region; S: subcutaneous abdominal; F: falciform ligament; * *p* < 0.05.

We used RT-PCR to evaluate the expression of AD-MSC surface marker genes (CD90, CD44, and CD29), hematopoietic markers (CD45 and CD34), and the pluripotent transcription factor OCT-4 in SVF cells from different harvesting sites. Our results showed that AD-MSC genes were expressed in SVF cells from all sites, but peri-ovarian SVF cells showed the highest expression level. SVF samples from all sites showed weak expressions for CD45 and CD34. The OCT-4 gene was mainly expressed in peri-ovarian and subcutaneous abdominal fats but weakly expressed in falciform ligament fats (Figure 6 and Figure 7).

## 4. Discussion

Adipose tissue is an appealing cell source for regenerative and engineering medicine due to easy harvesting and the abundance of stem cell populations. Autologous adipose SVF injection has gained popularity in the orthopedic field because it is a favorable, minimally invasive, and non-surgical alternative for the handling of musculoskeletal disorders [28]. Despite the increasing number of pre-clinical and clinical studies on the potential role of SVF cells to treat osteoarthritis and/or cartilage lesions, clear findings are missing due to the insufficient standardization of SVF cell isolation and characterization [29]. This study addressed the best AT sampling sites for SVF cell isolation in canines as an excellent model for humans. SVF cells were isolated from different AT sampling sites (subcutaneous abdominal, falciform ligament, and peri-ovarian fats). Then, we compared the isolated SVF cells regarding the cell viability and cellular yield using trypan blue staining and a hemocytometer, as well as AD-MSC surface markers’ expression by flow cytometry.

Our study results revealed that the peri-ovarian site is an excellent and suitable source for AT harvest, with a mean weight of 8.13 ± 4.3 gm, because ovariohysterectomy, as a routine surgery, is a relatively easy method to obtain high amounts of AT during surgery without significant risk for the donor’s tissues. This fact should be considered when the donor is emaciated or has chronic or nutritional diseases. However, subcutaneous abdominal AT harvests required more dissection and relied on higher body condition scores of the donor animals [20,30], which may explain the lower mean weight of subcutaneous abdominal AT harvests (2.7 ± 1.6 gm) in our study. Although a high AT weight (9.47 ± 2.9 gm) was harvested from the falciform ligament site in our study, the falciform AT collection may cause some complications, such as postoperative pain related to intra-abdominal adhesion, celiotomy, seroma, abdominal incision dehiscence, or incision site infection [31]. The mean weight of falciform AT samples in the current study was lower than that reported by Astor et al. [20] (91.42 ± 48.55 gm). This difference may be attributed to the large sample of client-owned dogs used in the study of Astor et al. [20]. The larger scale of falciform fat samples evaluated in the study of Sullivan et al. [21] may represent complete removal of the falciform ligament from euthanized dogs, while the small scale of the falciform sample collected from live dogs in the present study was aimed to conserve materials.

Site-specific properties of the adipose tissue plus paracrine interactions between adipose harvests and contiguous tissues have been considered in the previous studies with the perinodal AT around lymph nodes [32], perivascular AT [33], and pericardial AT [34]. The differences in cell isolation from various anatomical locations recorded in both the current study and the previous literature may be attributed to the different degrees of vascularization of AT and the harvest sites. Different canine breeds included in this study showed homogenous results related to the studied parameters. We did not include the body condition score of donors in this study due to its non-significant effect on the viable cells% per gram fat as previously proposed by Astor et al. [20]. Additionally, AD-MSC yield was not correlated with body mass index in humans [35].

Precise determination of cell viability and concentration in the freshly isolated adipose SVF is critical to accomplish the clinical research outcomes [1]. SVF cells isolated in this study from different sites showed a very good viability exceeding 90%. However, the highest SVF cell viability percentage was recorded from the peri-ovarian AT (99.63 ± 0.2%). These results disagreed with the study of DePompeo et al. [31], who reported a lower cell viability percent. The lower percentage of cell viability recorded by DePompeo et al. [31] may be related to sample storage for 20 h before tissue digestion, resulting in a 10–20% decrease in the viability.

Here, we examined the impact of AT harvesting sites on the number of viable cells per gram fat, which is essential for the presence of sufficient cells for treatment procedures. Among the examined sites, the peri-ovarian harvest showed the highest concentration of viable cells/gram of digested fat at *** *p*< 0.001. Moreover, a non-significant difference in viable cell number/gm fat was noticed between the subcutaneous abdominal and falciform ligament AT, which was consistent with the results of Guercio et al. [15]. By contrast, Astor et al. [20] reported that viable cells/gm of the SVF isolated from falciform AT were lower than that of the subcutaneous fat in spayed/neutered dogs. This disagreement could be explained by using non-spayed female donors in the present study, and Astor et al. [20] speculated that hormones may influence the viable cells per gram of tissue at the falciform location.

Adipose SVF has been used to treat multiple inflammatory and immune-mediated disorders in canines [10,11,18,19]. However, the presence of sufficient AD-MSCs, with a differentiation capacity, in SVF isolates is crucial for a successful treatment. Thus, the characterization of SVF cells is an important aspect of quality control for use in regenerative therapies. Flow cytometry can identify the different cell types within the adipose SVF [36]. Moreover, immunophenotyping is frequently achieved by flow cytometry to identify individual cells that simultaneously express the key MSC markers and lack the expression of hematopoietic markers. These cell surface and intracellular markers belong to the cluster of differentiation (CD) group [37]. To identify AD-MSCs, we labeled SVF samples with known stem cell markers. Although we could not define SVF cells with multiple MSC marker criteria due to the lack of suitable facilities, our results showed that the isolated SVF from different sites expressed AD-MSC surface markers (CD90, CD44, and CD29) and lacked the CD45 hematopoietic stem cell marker. This expression profile agreed with previous studies [24,25], showing that most AD-MSCs are CD90+, CD44+, CD29+ and CD45−. Positive markers consist of members of the integrin family, such as CD90 surface marker, which present on a high proportion of MSCs (71.4% ± 15.8%). The activation of CD90 stimulates T cell activation in addition to the regulation of various biological mechanisms, such as cell–cell and cell–matrix cellular interactions in axon regeneration, adhesion, apoptosis, migration, fibrosis, and cancer [38]. CD29, the Very Late Activation antigen, participates in the mechanism of cell adhesion [39]. Another MSC positive marker is the hyaluronate receptor CD44, which is a non-integrin cell surface marker essential for the adhesion of different leukocytes to endothelia and T-lymphocyte activation [40].

We studied the effect of the AT harvesting site in canines on the potential yield of AD-MSCs in the isolated SVF. The mean percentage of CD90+ viable cells in this study varied between samples from different harvesting sites. Cells isolated from the peri-ovarian site showed the highest CD90+ expression (49.56 ± 4.9%), with a significant difference. Similarly, the highest proportion of CD44 + cells was recorded in the peri-ovarian SVF cells. These data were significantly different from the falciform ligament site but not from the subcutaneous abdominal site. Although CD29+ve cells showed no significant differences among the three sites, the peri-ovarian SVF cells had the highest CD29+ mean percentage (38.00 ± 7.62%). CD45 negative cells from the subcutaneous abdominal and peri-ovarian sites were nearly equal and significantly higher than those from the falciform ligament site, indicating a lower contamination with hematopoietic cells in the first two sites. Although the falciform CD45− cells’ mean percentage in our study approached that of Sulvian et al. [21], our CD90+ and CD44+ cells were lower than those of Sulvian et al. [21]. This difference is possibly due to the variation in SVF cell isolation protocols and in the donors’ ages. Quaade et al. [12] observed that younger rats had more MSC cells in SVF than aged ones, suggesting that the animal age affected cell type relative distribution in the SVF cell population. Here, we did not cultivate SVF to address the differentiation potential because we mainly focused on studying the freshly isolated uncultivated SVF as a point-of-care therapy. Culturing SVF cells for even one passage would alter their cellular composition and differentiation potential [41]. Additionally, our cryopreserved SVF samples were not suitable for assessing the differential potential according to Duan and Lopez [42], who reported that cryopreservation alters AD-MSCs ultrastructure and immunophenotype. Further studies are needed to evaluate the therapeutic effects of SVF cells isolated from different sites on musculoskeletal problems. However, our flow cytometry data showed that cells isolated from the peri-ovarian site had an AD-MSC concentration higher than that from the falciform ligament and subcutaneous abdominal sites.

We used RT-PCR to evaluate the gene expression of the same markers analyzed by flow cytometry as well as Oct-4 and CD34. The RT-PCR results agreed with results obtained by flow cytometry. SVF cells from the peri-ovarian site maintained stable expression of MSC markers (CD90, CD44, and CD29) and pluripotent transcription factor OCT-4 in a higher level compared to the other sites. The positive expression of Oct-4 demonstrated the pluripotency of AD-MSC as well as it has already been described in canines [26]. The core pluripotent transcription factors, such as Oct-4, Sox-2, and Nanog, regulate the self-renewal ability and differentiation abilities of AD-MSCs [43]. SVF cells from all sites revealed weak expression of hematopoietic markers (CD45 and CD34). CD34 is a physiological niche-specific marker of immature/early progenitor cells, which is lost in the in vitro condition. CD34 marks different progenitor cell types, such as different MSCs and vascular endothelial progenitor cells [29]. Together, our flow cytometry and RT-PCR results suggest that the peri-ovarian site AT harvest site may have a higher potential for use in regenerative therapy.

## 5. Conclusions

The peri-ovarian AT harvesting site is a favorable and suitable source for AT harvest in middle-aged and old dogs, without substantial risk to the donor tissues. This harvesting site could be valuable in emaciated donors. The peri-ovarian harvesting site yielded a higher SVF viability percentage, viable cell number/gm fat and AD-MSC marker expression than that of the other harvesting sites, indicating a high potential for application in regenerative therapy. This study may lay the foundation for further studies in the set-up of cell-based regenerative therapies at the preclinical and experimental level in the canine model.

## Figures and Tables

**Table 1:** The list of antibodies used for flow cytometry.

Cell Surface Marker	Antibody Clone	Species Reactivity	Clonality	Antibody Quantity	Cat. No	Source
CD90 PE	5E10	Dog	Monoclonal	10 µL/10^6^ cells	ARG54208	Arigo Biolaboratories
CD44 PE	IM7	Dog	Monoclonal	10 µL/10^6^ cells	GTX80086	GeneTex
CD29 PE	MEM-101A	Dog	Monoclonal	10 µL/10^6^ cells	1P219T025	EXBIO antibodies
CD45 FITC	YKIX716.13	Dog	Monoclonal	10 µL/10^6^ cells	GTX43583	GeneTex

CD: Cluster of Differentiation, PE: Phycoerythrin, FITC: fluorescein isothiocyanate.

**Table 2:** Primers used in RT-PCR.

Target Gene	Accession Number	Primers	Amplicon Size	Reference
Oct-4	XM_538830.1	Fw: AAGCCTGCAGAAAGACCTGRv: GTTCGCTTTCTCTTTCGGGC	286 bp	Ivanovska et al. [26]
CD90	NM_001287129.1	Fw: AAGCCAGGATTGGGGATGTGRv: TGTGGCAGAGAAAGCTCC TG	285 bp	Ivanovska et al. [26]
CD44	NM_001197022.1	Fw: CCCATTACCAAAGACCACGARv: TTCTCGAGGTTCCGTGTCTC	408 bp	Ivanovska et al. [26]
CD29	XM_005616949.1	Fw: AGGATGTTGACGACTGCTGGRv: ACCTTTGCATTCAGTGTTGTGC	356 bp	Ivanovska et al. [26]
CD45	XM_005622282.1	Fw: TGTTTCCAGTTCTGTTTCCCCARv: TCAGGTACAAAGCCTTCCCA	432 bp	Ivanovska et al. [26]
CD34	NM_001003341.1	Fw: GAGATCACCCTAACGCCTGGRv: GGCTCCTTCTCACACAGGAC	383 bp	Ivanovska et al. [26]
*β*-actin	XM_544346	Fw: GAGACCTGACCGACTACCTRv: GCT GCCTCCAGACAACAC	553 bp	Qiu et al. [27]

**Table 3:** Data of animals used in adipose tissue harvesting.

Breed	Body Weight (Kg)	Age (Years)	Sex	Adipose Tissue Harvest (gm)	Viability%	Viable Cell/Gram × 10^6^
Beagle	9	8	Female	1 S	99.8	0.24
				7.3 F	90	4.2
				9.5 P	99.8	46.2
Beagle	10	8	Female	1.3 S	92	2.4
				14.7 F	90.9	3
				2 P	99.7	24
Chihuahua	3.7	9	Female	1 S	96.6	27.5
				8 F	98.7	5.4
				2.5 P	99.8	28
Chihuahua	6.14	8	Female	5.5 S	95.5	0.43
				6.5 F	90.9	8.95
				2.6 P	99.6	29
Miniature Dachshund	7.7	10	Female	4.7 S	97.1	2
				10 F	97.9	3.3
				7 P	99.7	44.6
Pug	6	8	Female	1 S	95.2	2.4
				8.3 F	95	3.6
				6 P	99.6	31
Golden Retriever	20.6	8	Female	3 S	97.2	1.6
				10 F	98.4	8
				12 P	99.3	87.5
Jack Russell Terrier	9.72	10	Female	2.6 S	89.7	4.2
				8.2 F	98.8	1.6
				2 P	99.7	19.6
Pomeranian	8	11	Female	3.4 S	93.4	0.78
				14.7 F	97	8.1
				14 P	99.8	32.8
Mix	14.7	9	Female	4.2 S	92.9	0.29
				7 F	88.2	11
				5.8 P	99.2	26

S: subcutaneous abdominal; F: falciform ligament; P: peri-ovarian region.

## Data Availability

Data are contained within the article.

## References

[1] Chan L.L.-Y., Cohen D.A., Kuksin D., Paradis B.D., Qiu J. (2014). Automated enumeration and viability measurement of canine stromal vascular fraction cells using fluorescence-based image cytometry method. J. Fluoresc..

[2] Mohal J.S., Tailor H.D., Khan W.S. (2012). Sources of adult mesenchymal stem cells and their applicability for musculoskeletal applications. Curr. Stem Cell Res. Ther..

[3] Nakao N., Nakayama T., Yahata T., Muguruma Y., Saito S., Miyata Y., Yamamoto K., Naoe T. (2010). Adipose tissue-derived mesenchymal stem cells facilitate hematopoiesis in vitro and in vivo: Advantages over bone marrow-derived mesenchymal stem cells. Am. J. Pathol..

[4] Choudhery M.S., Badowski M., Muise A., Harris D.T. (2013). Comparison of human mesenchymal stem cells derived from adipose and cord tissue. Cytotherapy.

[5] Zuk P.A., Zhu M., Mizuno H., Huang J., Futrell J.W., Katz A.J., Benhaim P., Lorenz H.P., Hedrick M.H. (2001). Multilineage cells from human adipose tissue: Implications for cell-based therapies. Tissue Eng..

[6] Marx C., Silveira M.D., Nardi N.B. (2015). Adipose-derived stem cells in veterinary medicine: Characterization and therapeutic applications. Stem Cells Dev..

[7] Markarian C.F., Frey G.Z., Silveira M.D., Milani A.R., Ely P.B., Horn A.P., Nardi N.B., Camassola M. (2014). Isolation of adipose-derived stem cells: A comparison among different methods. Biotechnol. Lett..

[8] Yoshimura K., Shigeura T., Matsumoto D., Sato T., Takaki Y., Aiba-Kojima E., Sato K., Inoue K., Nagase T., Koshima I. (2006). Characterization of freshly isolated and cultured cells derived from the fatty and fluid portions of liposuction aspirates. J. Cell. Physiol..

[9] De Girolamo L., Lucarelli E., Alessandri G., Antonietta Avanzini M., Ester Bernardo M., Biagi E., Teresa Brini A., D’Amico G., Fagioli F., Ferrero I. (2013). Mesenchymal stem/stromal cells: A new’ cells as drugs paradigm. Efficacy and critical aspects in cell therapy. Curr. Pharm. Des..

[10] Marx C., Silveira M.D., Selbach I., Da Silva A.S., Braga L.M.G.D.M., Camassola M., Nardi N.B. (2014). Acupoint injection of autologous stromal vascular fraction and allogeneic adipose-derived stem cells to treat hip dysplasia in dogs. Stem Cells Int..

[11] Upchurch D.A., Renberg W.C., Roush J.K., Milliken G.A., Weiss M.L. (2016). Effects of administration of adipose-derived stromal vascular fraction and platelet-rich plasma to dogs with osteoarthritis of the hip joints. Am. J. Vet. Res..

[12] Quaade M.L., Jensen C.H., Andersen D.C., Sheikh S.P. (2016). A 3-month age difference profoundly alters the primary rat stromal vascular fraction phenotype. Acta Histochem..

[13] Kemilew J., Sobczyńska-Rak A., Żylińska B., Szponder T., Nowicka B., Urban B. (2019). The use of allogenic stromal vascular fraction (SVF) cells in degenerative joint disease of the spine in dogs. In Vivo.

[14] Lee K.S., Kang H.W., Lee H.T., Kim H.-J., Kim C.-L., Song J.-Y., Lee K.W., Cha S.-H. (2014). Sequential sub-passage decreases the differentiation potential of canine adipose-derived mesenchymal stem cells. Res. Vet. Sci..

[15] Guercio A., Di Bella S., Casella S., Di Marco P., Russo C., Piccione G. (2013). Canine mesenchymal stem cells (MSCs): Characterization in relation to donor age and adipose tissue-harvesting site. Cell Biol. Int..

[16] Bergknut N., Rutges J.P., Kranenburg H.J.C., Smolders L.A., Hagman R., Smidt H.J., Lagerstedt A.S., Penning L.C., Voorhout G., Hazewinkel H.A. (2012). The dog as an animal model for intervertebral disc degeneration?. Spine.

[17] McMahill B.G., Borjesson D.L., Sieber-Blum M., Nolta J.A., Sturges B.K. (2015). Stem cells in canine spinal cord injury-promise for regenerative therapy in a large animal model of human disease. Stem Cell Rev. Rep..

[18] Albano D., Messina C., Usuelli F.G., De Girolamo L., Grassi M., Maccario C., Bignotti B., Tagliafico A., Sconfienza L.M. (2017). Magnetic resonance and ultrasound in Achilles tendinopathy: Predictive role and response assessment to platelet-rich plasma and adipose-derived stromal vascular fraction injection. Eur. J. Radiol..

[19] Abdallah A.N., Shamaa A.A., El-Tookhy O.S. (2019). Evaluation of treatment of experimentally induced canine model of multiple sclerosis using laser activated non-expanded adipose derived stem cells. Res. Vet. Sci..

[20] Astor D.E., Hoelzler M.G., Harman R., Bastian R.P. (2013). Patient factors influencing the concentration of stromal vascular fraction (SVF) for adipose-derived stromal cell (ASC) therapy in dogs. Can. J. Vet. Res..

[21] Sullivan M.O., Gordon-Evans W.J., Fredericks L.P., Kiefer K., Conzemius M.G., Griffon D.J. (2016). Comparison of mesenchymal stem cell surface markers from bone marrow aspirates and adipose stromal vascular fraction sites. Front. Vet. Sci..

[22] Watkins S., Hall L., Clarke K. (1987). Propofol as an intravenous anaesthetic agent in dogs. Vet. Rec..

[23] Tomihari M., Nishihara A., Shimada T., Yanagawa M., Miyoshi M., Miyahara K., Oishi A. (2015). A comparison of the immunological effects of propofol and isoflurane for maintenance of anesthesia in healthy dogs. J. Vet. Med. Sci..

[24] Krešić N., Šimić I., Lojkić I., Bedeković T. (2017). Canine adipose derived mesenchymal stem cells transcriptome composition alterations: A step towards standardizing therapeutic. Stem Cells Int..

[25] Yaneselli K.M., Kuhl C.P., Terraciano P.B., De Oliveira F.S., Pizzato S.B., Pazza K., Magrisso A.B., Torman V., Rial A., Moreno M. (2018). Comparison of the characteristics of canine adipose tissue-derived mesenchymal stem cells extracted from different sites and at different passage numbers. J. Vet. Sci..

[26] Ivanovska A., Grolli S., Borghetti P., Ravanetti F., Conti V., De Angelis E., Macchi F., Ramoni R., Martelli P., Gazza F. (2017). Immunophenotypical characterization of canine mesenchymal stem cells from perivisceral and subcutaneous adipose tissue by a species-specific panel of antibodies. Res. Vet. Sci..

[27] Qiu C., Lin D., Wang H., Qiao C., Wang J., Zhang T. (2008). Quantification of VEGF-C expression in canine mammary tumours. Aust. Vet. J..

[28] Senesi L., De Francesco F., Farinelli L., Manzotti S., Gagliardi G., Papalia G.F., Riccio M., Gigante A. (2019). Mechanical and enzymatic procedures to isolate the stromal vascular fraction from adipose tissue: Preliminary results. Front. Cell Dev. Biol..

[29] Bora P., Majumdar A.S. (2017). Adipose tissue-derived stromal vascular fraction in regenerative medicine: A brief review on biology and translation. Stem Cell Res. Ther..

[30] Zhang N., Dietrich M.A., Lopez M.J. (2013). Canine intra-articular multipotent stromal cells (MSC) from adipose tissue have the highest in vitro expansion rates, multipotentiality, and MSC immunophenotypes. Vet. Surg..

[31] DePompeo C.M., Giassetti M.I., Elnaggar M.M., Oatley J.M., Davis W.C., Fransson B.A. (2020). Isolation of canine adipose-derived mesenchymal stem cells from falciform tissue obtained via laparoscopic morcellation: A pilot study. Vet. Surg..

[32] Pond C.M., Mattacks C.A. (1995). Interactions between adipose tissue around lymph nodes and lymphoid cells in vitro. J. Lipid Res..

[33] Löhn M., Dubrovska G., Lauterbach B., Luft F.C., Gollasch M., Sharma A.M. (2002). Periadventitial fat releases a vascular relaxing factor. FASEB J..

[34] Fox C.S., Gona P., Hoffmann U., Porter S.A., Salton C.J., Massaro J.M., Levy D., Larson M.G., D’Agostino R.B., O’Donnell C.J. (2009). Pericardial fat, intra-thoracic fat, and measures of left ventricular structure and function: The Framingham heart study. Circulation.

[35] Jurgens W.J., Oedayrajsingh-Varma M.J., Helder M.N., Zandieh Doulabi B., Schouten T.E., Kuik D.J., Ritt M.J., Van Milligen F.J. (2008). Effect of tissue-harvesting site on yield of stem cells derived from adipose tissue: Implications for cell-based therapies. Cell Tissue Res..

[36] Dubey N.K., Mishra V.K., Dubey R., Deng Y.-H., Tsai F.-C., Deng W.-P. (2018). Revisiting the advances in isolation, characterization and secretome of adipose-derived stromal/stem cells. Int. J. Mol. Sci..

[37] Sasaki A., Mizuno M., Mochizuki M., Sekiya I. (2019). Mesenchymal stem cells for cartilage regeneration in dogs. World J. Stem Cells.

[38] Hagood J.S. (2019). Thy-1 as an integrator of diverse extracellular signals. Front. Cell Dev. Biol..

[39] Harjunpää H., Asens M.L., Guenther C., Fagerholm S.C. (2019). Cell adhesion molecules and their roles and regulation in the immune and tumor microenvironment. Front. Immunol..

[40] Li J., Zanata F., Curley J.L., Martin E.C., Wu X., Dietrich M., Devireddy R.V., Wade J.W., Gimble J.M. (2016). The relative functionality of freshly isolated and cryopreserved human adipose-derived stromal/stem cells. Cells Tissues Organs.

[41] Sun Y., Chen S., Zhang X., Pei M. (2019). Significance of cellular cross-talk in stromal vascular fraction of adipose tissue in neovascularization. Arter. Thromb. Vasc. Biol..

[42] Duan W., Lopez M.J. (2016). Effects of cryopreservation on canine multipotent stromal cells from subcutaneous and infrapatellar adipose tissue. Stem Cell Rev. Rep..

[43] Reich C.M., Raabe O., Wenisch S., Bridger P.S., Kramer M., Arnhold S. (2012). Isolation, culture and chondrogenic differentiation of canine adipose tissue and bone marrow-derived mesenchymal stem cells–A comparative study. Vet. Res. Commun..

